# Genetic variants of chemokine receptor CCR7 in patients with systemic lupus erythematosus, Sjogren's syndrome and systemic sclerosis

**DOI:** 10.1186/1471-2156-8-33

**Published:** 2007-06-22

**Authors:** Daniel Kahlmann, Ana Clara Marques Davalos-Misslitz, Lars Ohl, Frauke Stanke, Torsten Witte, Reinhold Förster

**Affiliations:** 1 Institute of Immunology, Hannover Medical School, 30625 Hannover, Germany; 2 Deparment for Pediatric Pneumology Hannover Medical School, 30625 Hannover, Germany; 3 Department for Clinical Immunology, Hannover Medical School, 30625 Hannover, Germany

## Abstract

**Background:**

The chemokine receptor CCR7 is a key organizer of the immune system. Gene targeting in mice revealed that Ccr7-deficient animals are severely impaired in the induction of central and peripheral tolerance. Due to these defects, Ccr7-deficient mice spontaneously develop multi-organ autoimmunity showing symptoms similar to those observed in humans suffering from connective tissue autoimmune diseases. However, it is unknown whether mutations of *CCR7 *are linked to autoimmunity in humans.

**Results:**

DNA samples were collected from 160 patients suffering from connective tissue autoimmune disease (Sjogren's syndrome, n = 40; systemic lupus erythematosus, SLE, n = 20 and systemic sclerosis, n = 100) and 40 health subjects (n = 40). All participants in this study were of German descent. Samples were screened for single nucleotide polymorphisms (SNP) by sequencing the coding region of the *CCR7 *gene as well asthe exon flaking intron sites and parts of the regions encoding for the 5'- and 3'-UTR. *CCR7 *variants were rare. We identified six different sequence variants, which occurred in heterozygosis. The identified SNP were observed at position -60 C/T (observed 1x), +6,476 A/G (7x), +6,555 C/T (15x), +6,560 C/T (6x), +10,440 A/G (3x) and +11,475 C/A (1x). Four of these variants (+6,476 A/G, +6,555 C/T, +6,560 C/T and +10,440 A/G) display allelic frequencies between 1% and 5 % and were present in both patients and control groups. The variants +6,476 A/G, +6,555 C/T, +6,560 C/T are located in the intron 2, while the +10,440 A/G variant corresponds to a silent mutation in exon 3. The variants -60 C/T and +11,475 C/A which are located at the 5'-UTR and 3-UTR respectively, display allelic frequencies below 1%. No correlation between these variants and the autoimmune diseases investigated could be observed. However, reporter gene expression assay demonstrated that the mutation at the -60 C/T position in homozygosis leads to reduced luciferase activity.

**Conclusion:**

These results suggest that variants of *CCR7 *gene occur at an extremely low frequency in the German population and that neither Sjogren's syndrome, systemic lupus erythematosus, nor systemic sclerosis are associated with these variants. Nevertheless, the decreased luciferase activity observed in cells transfected with the promoter region bearing the -60 C/T mutation suggests that this *CCR7 *variant could potentially lead to increased susceptibility to autoimmunity.

## Background

The functional organization of the immune system relies on the orchestrated migration of its cellular components. Along with adhesion molecules, chemokines and their receptors play an essential role in directing the complex network of continuous cell trafficking, homing to lymphoid and non-lymphoid organs and egressing from these sites [1]. The CC-chemokine receptor 7 (CCR7) and its ligands, CCL19 and CCL21, have been identified as one of the key players in lymphocyte and dendritic cell migration [2,3]. Ccr7 is required for the homing of naïve T cells via high endothelial venules (HEV) to lymph nodes and Peyer's patches, the correct positioning of B cells and T cells within secondary lymphoid organs, as well as the egress of dendritic cells from the skin to the draining lymph nodes under both steady state and inflammatory conditions [2,4]. Furthermore, together with Cxcr5, Ccr7 is required for the development of lymph nodes and Peyer's patch by guiding lymphoid tissue inducing cells to lymph node anlagen [5]. Consequently, gene-targeted mice deficient in Ccr7 show profound alterations of the immune system that result in delayed and impaired immune responses [2,4].

We have recently demonstrated that ccr7-deficient mice show gross alterations in thymus architecture with loss of the classical cortex-medulla segregation [6]. This defect seems to be due to impaired migration of early T cell progenitors from the cortico-medullary junction towards the subcapsular regions. Others have suggested that positively selected thymocytes need ccr7 to migrate from the cortex into the medulla [7]. These migration defects result in breakdown of central tolerance. Consequently, Ccr7-deficient mice show prominent cellular infiltrates in several organs including salivary and lachrymal glands, stomach, pancreas and liver [7,8]. In addition, Ccr7^-/- ^mice display increased titers of circulating auto-antibodies to nuclear and non-nuclear tissue-antigens as well as IgG deposits on renal glomeruli. Ccr7-deficient mice show increased susceptibility to streptozotocin-induced diabetes as well as manifestations of ongoing spontaneous autoimmune renal disease [8]. Since Ccr7 deficiency in mice results in morphological and functional alterations, which are also characteristic for various human autoimmune diseases, we sequenced part of the *CCR7 *locus in 160 autoimmune patients and 40 healthy controls.

## Results

### Analysis of the *CCR7 *sequences

We recently demonstrated that Ccr7-deficient mice are prone to develop generalized multi-organ autoimmunity and spontaneously display symptoms of human connective tissue autoimmune diseases [8]. To investigate a possible correlation between *CCR7 *variants and susceptibility to the development of autoimmune diseases in humans, we performed a sequencing analysis of the *CCR7 *gene in 100 patients suffering from systemic sclerosis, in 40 patients with Sjogren's syndrome and in 20 patients suffering from SLE. Disease classification criteria of the investigated patients are shown in Table 1. Age-matched healthy individuals served as controls. Both patients and controls were of German descent. We investigated *CCR7 *variants by direct sequencing of its coding sequence, flaking introns sites (accounting for splice variants) and the core promoter, encompassing *in toto *3 kb. This approach allows the identification of mutations in the coding sequence as well as in regulatory elements which could influence CCR7 expression.

**Table 1:** Disease classification criteria

**Disease**	**Criteria**	**% of patients fulfilling the criteria**
SLE	Malar rash	90
	Discoid rash	55
	Photosensitivity	70
	Oral ulcerations	40
	Non-erosive arthritis	90
	Serositis	35
	Glomerulonephritis	40
	Neurological involvement	15
	Hematological involvement	65
	Immunological involvement	75
	Antinuclear antibodies	100

Sjogren's syndrome	Ocular symptoms	95
	Oral symptoms	87,5
	Objective involvement of lacrimal glands	92,5
	Pathological salivary gland biopsy	32,5
	Objective involvement of salivary glands	75
	Presence of autoantibodies against Ro/La	90

systemic sclerosis	Proximal scleroderma	100
	Sclerodactyly	44
	Acral ulcerations	36
	Bilateral pulmonary fibrosis	18

Patients analyzed in the present study have been classified based on the listed criteria.

**Figure 1:**
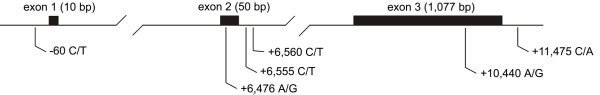
Schematic overview of the *CCR7 *gene located at chromosome 17q12 – q21.2. The enlarged areas represent the three exons with their flanking regions that have been subjected to sequence analysis. The positions of the observed SNP are given in relation to the first base of exon 1 which has been set to +1.

We identified six different sequence variants which occurred heterozygously. Since the transcriptional starting point of the *CCR7 *gene has not yet been mapped, the location of the variants is given relative to the position of the first base coding for the methionine of exon 1 which was set to position +1 and the preceding base to -1. Six SNP were identified at positions -60 C/T (observed 1x), +6,476 A/G (7x), +6,555 C/T (15x), +6,560 C/T (6x), +10,440 A/G (3x) and +11,475 C/A (1x; see Table 2 and Fig. 1). All genotype frequencies were consistent with Hardy-Weinberg distribution for all variants analyzed and in all patient and control cohorts (p < 0.05).

**Table 2:** Location and frequency of the observed SNP

**Position**	**Location**	**SNP**	**Exchange in amino acid?**	**Population**	**Number of subjects carrying the SNP**	**Number of analyzed subjects**	**Allelic frequency (%)**	**Reference**
-60	5'-UTR	C/T		Systemic sclerosis	1	100	0.50	this work
				SLE	0	20	0.00	
				Sjogren's syndrome	0	40	0.00	
				Controls	0	40	0.00	

+6,476	exon 2	A/G	methionine to valine	Systemic sclerosis	4	100	2.00	this work
				SLE	0	20	0.00	
				Sjogren's syndrome	1	40	1.25	
				Controls	2	40	2.50	

+6,555	intron 2	C/T		Systemic sclerosis	6	100	3.00	this work
				SLE	2	20	5.00	
				Sjogren's syndrome	3	40	3.75	
				Controls	4	40	5.00	

+6,560	intron 2	C/T		Systemic sclerosis	4	100	2.00	rs3136689
				SLE	0	20	0.00	(HapMap)
				Sjogren's syndrome	0	40	0.00	
				Controls	2	40	2.50	

+10,440	exon 3	A/G	no exchange	Systemic sclerosis	2	100	1.00	rs2229095
				SLE	0	20	0.00	(HapMap)
				Sjogren's syndrome	1	40	1.25	
				Controls	0	40	0.00	

+11,475	3'-UTR	C/A		Systemic sclerosis	1	100	0.50	this work
				SLE	0	20	0.00	
				Sjogren's syndrome	0	40	0.00	
				Controls	0	40	0.00	

Positions of the observed SNP are given relative to the first base coding for the Methionine of exon 1 which has been set to position +1 and the preceding base to -1. SNP, single nucleotide polymorphism. The most frequent allele is named first.

Four out of six identified SNP (-60 C/T, +6,476 A/G, +6,555 C/T and +11,475 C/A) have not been described so far. The variants +6,560 C/T and +10,440 A/G were previously described in the HapMap SNP database and correspond to the rs3136689 and rs2229095 SNP, respectively (Table 2). Only two of the observed variants (+6,476 A/G and +10,440 A/G) were located within the *CCR7 *coding sequence (Table 2 and Fig. 1). The +6,476 A/G variant at exon 2 resulted in an exchange at the 1^st ^base of the ATG-codon which in turn resulted in a methionine to valine exchange. This mutation did not correlated with increased susceptibility to the diseases investigated here since similar frequencies were observed in patients and controls (Table 2). The +10,440 A/G variant (rs2229095, HapMap) of exon 3 was not observed in the control group (Table 2). However, as this mutation resulted in a silent transversion at the 3^rd ^base of codon CAA, this variant is also likely to be irrelevant for susceptibility to connective tissue autoimmune diseases. In addition, the frequency of the allele G observed in the patient group (1% in patients suffering from systemic sclerosis and 1.25% in patients suffering from Sjogren's syndrome (Table 2), was similar to the allelic frequency (0.8%) previously observed in Utah residents with ancestry from northern and western Europe (CEU HapMap samples, n = 60). The mutations +6,555 C/T and +6,560 C/T (rs3136689; HapMap) were located in intron 2. The +6,555 C/T SNP was observed in all patients and control groups at similar frequencies (Table 2). In contrast, the rs3136689 SNP was only observed in patients suffering from systemic sclerosis and in the control individuals. The allelic frequency of this SNP was similar in both groups (2.0% and 2.5%, respectively; Table 2). Interestingly, the frequency of the allele T observed in the CEU population differs from that observed in our control samples (0.8% *vs*. 2.5%, respectively, Table 2). It is however of note that our control group consists of healthy German subjects without signs of autoimmune diseases and tested negative for the above mentioned diseases. Therefore, it seems likely that the discrepancies observed between the CEU population and our control group are a consequence of the small size of the compared samples (n = 60 and n = 40, respectively). The variants -60 C/T and +11,475 C/A were located in the 5'-UTR and 3'-UTR respectively. Both mutations occurred at a very low allelic frequency (0.5%) and were only detected in the group of patients suffering from systemic sclerosis (Table 2). The absence of these rare variants in the other groups likely reflects the small size of the samples rather than a correlation between these alleles and susceptibility to systemic sclerosis. Nevertheless, mutations in such regulatory regions of the gene could lead to decreased CCR7-expression and therefore increases the susceptibility of individual subjects to develop autoimmune disease.

In summary, *CCR7 *variants were rare among patients and controls with allelic frequencies always ≤ 5%. In addition, no clear correlation has been found between autoimmunity and the observed *CCR7 *variants. Furthermore, the results largely exclude the possibility that rare sequence variants within the coding sequences, flanking intron sites and the *CCR7 *core promoter accumulate in subjects suffering from autoimmune diseases investigated in this study.

### The -60 C/T mutation leads to reduced reporter gene expression *in vitro*

**Figure 2:**
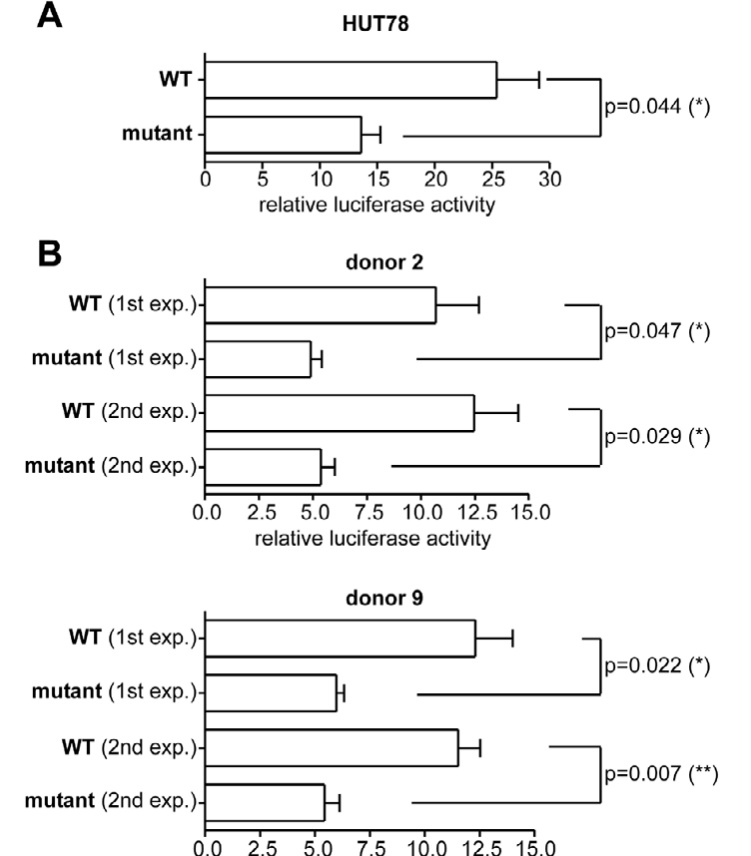
Relative luciferase activity of the wild type promoter (WT) and a promoter construct bearing the -60 C/T mutation in homozygosis. (A): relative luciferase activity in HUT78 cells following transfection of the promoter constructs by electroporation. Data shown is representative of three independent experiments. (B): relative luciferase activity in primary T-cells that were transfected with the promoter constructs using Amaxa transfection technology. Two independent experiments (exp.) were performed. In each experiment primary T-cells from 13 donors were used. Shown are results from donor #2 and donor #9. No differences between WT and mutant promoter activity were observed in the remaining 11 donors. Error bars represent the SEM of triplicates. Statistic analysis was performed applying unpaired t-test. Values < 0.05 were considered significant (* = p < 0.05, ** = p < 0.01).

Since the exchange at position -60 might affect the *CCR7 *promoter activity, we further characterized this variant. *In silico *analysis revealed a putative binding site for AP-2/Ikaros at position -77 to -47. To functionally test whether this variant has any effect on CCR7 expression, we cloned a 563 bp fragment of the wild type and the variant promoter into a firefly luciferase reporter plasmid. To adjust for transfection efficiency, each construct was co-transfected with a renilla luciferase reporter plasmid. Promoter activity has been tested by transfecting the T cell line HUT78 using a conventional electroporation protocol. As shown in Fig. 2A the relative luciferase activity of HUT78 cells transfected with the construct bearing the mutant promoter was roughly decreased twofold in comparison to that of cells transfected with the construct bearing the wild type promoter. This result indicates that the -60 C/T mutation leads to a reduction of the *CCR7 *promoter activity. However, we can not currently rule out the possibility that this mutation has translational effects secondary to mRNA structural differences or mRNA stability. In order to compare the function of the wild type and mutant promoters in a more appropriated system taking into account the variability found normally in human populations, we transfected primary T-cells isolated from the peripheral blood of 13 unrelated healthy donors using Amaxa transfection technology. Under these experimental conditions we found that in 2 of the 13 donors the -60 C/T exchange also significantly reduced the *CCR7 *promoter activity (Fig. 2B) while no difference could be detected in the 11 remaining donors (data not shown). From the two donors that showed reduced promoter activity, blood was recollected 2 to 4 weeks later and the transfection experiment was repeated giving similar results (Fig. 2B). Since the results obtained by the transfection of primary cells are likely to reflect the situation observed in human populations more reliably than those obtained by the transfection of the HUT78 cell line, our data indicate that subject-specific mechanisms might influence the expression of CCR7 in T cells. Therefore, the -60 C/T mutation in combination with yet unknown genetic or environmental factors could potentially increase the susceptibility of individual persons to develop autoimmunity.

## Discussion

CCR7 is known as a key regulator of the immune system. Initial studies demonstrated that this receptor is required for the homing of naïve T cells into lymph node and Peyer's patches [2]. More recently we showed that dendritic cells in lung and intestine require Ccr7 to actively transport inhaled and ingested antigen into the draining lymph nodes of these organs in order to induce peripheral tolerance towards innocuous antigen [9,10]. Furthermore, Ccr7 expression is required for maintenance of the thymic architecture and appropriate negative selection (ref. [6] and unpublished data ACD-M and RF). Due to these defects, Ccr7-deficient mice are severely impaired in maintaining both peripheral and central tolerance [8-10]. Based on data derived from Ccr7-deficient mice we tested the hypothesis that mutations in the *CCR7 *gene might be associated with the development of autoimmune diseases in humans. However, data derived from 160 patients suffering from connective tissue autoimmune diseases and 40 healthy individuals revealed that genomic variants in the analyzed regions of the *CCR7 *gene locus occur at a very low frequency in the German population. In addition, the allelic frequencies of the identified variants were very low in all of the cohorts analyzed. These data demonstrate that mutations in the *CCR7 *gene do not positively correlate with development of the investigated autoimmune diseases. Interestingly, one of the identified mutations was located into the promoter region of the *CCR7 *gene. The activity of the mutant promoter was assessed by transient transfection of constructs bearing the luciferase gene into either HUT78 T cells or into primary T-cells. Luciferase expression driven by the mutant promoter was reduced in HUT78 cells to approximately 50% in comparison to that driven by the wild type promoter. Similar results were observed for primary T cells of 2 out of 13 donors analyzed. These data suggest that yet unknown factors can modulate the expression of CCR7 varying from one subject to another. This leaves the possibility open that rare mutations occurring in regulatory regions of the *CCR7 *gene could result in increased susceptibility to the development of autoimmune diseases in individual subjects.

## Methods

### Patients

Blood samples of patients with connective tissue diseases were collected in a study on genetic risk factors of autoimmune diseases approved by the local ethical committee. All patients were visiting the outpatient clinics of the Department of Clinical Immunology of Hannover Medical School, Germany. The patients with the connective tissue diseases SLE (n = 20, 85 % female), systemic sclerosis (n = 100, 80 % female) and primary Sjogren's syndrome (n = 40, 90 % female) were all German Caucasians from the area of Hannover and were classified according to the respective classification criteria [11-13]. Disease classification criteria of the analyzed patients are shown in Table 1. None of the patients with Sjögren's syndrome fulfilled the criteria of SLE or systemic sclerosis. Since in the absence of complaints of dry eyes or mouth a salivary gland biopsy was not performed, it cannot be ruled out that some of the patients with SLE or systemic sclerosis may have suffered from secondary Sjogren's syndrome. In addition, 40 isolates of healthy German Caucasian subjects without signs of the above-mentioned autoimmune diseases served as control. The average age of the patients was 45.9 +- 13.0 years and of controls 42.8 +- 12.1 years (mean +/- SD). Following informed written consent, blood samples were collected from each individual and genomic DNA was extracted from blood using standard procedures.

### DNA isolation and PCR

Genomic DNA was prepared from peripheral blood cells using the *QIAamp DNA Blood Mini Kit *(Qiagen). The coding regions as well as flanking regions of all three exons of the *CCR7 *gene were amplified using the primer pairs shown in Table 3. The PCR reactions were performed in 50 μl buffer (buffer Y, peqLab) containing 100 ng of human genomic DNA, 50 picomol of each primer, 2.5 nmol of each deoxyribonucleoside triphosphate, and 1.5 units of Taq polymerase. PCR reactions were performed for 40 cycles consisting of 8 seconds at 94°C, 30 seconds at 60°C and 90 seconds at 72°C following an initial denaturation step of 4 minutes at 95°C. For sequencing, PCR products were purified using Microcon YM-100 tubes (Milian, Italy). In some experiments PCR products were purified using the *QIAquick gel extraction kit*, (QIAgen).

**Table 3:** Primer used

**Primer**	**Sequence**	**Primer application**
CCR7_1F	GAT CCT ATG ACC AGC GAC TGT C	Forward amplification primer exon 1
		Sequencing primer exon 1
CCR7_1R	AGT AGC TTC CAA TGC CCA CCA AA	Reverse amplification primer exon 1
CCR7_2F	TAC CCC ACG ACC TCA TAG C	Forward amplification primer exon 2
CCR7_2R	GTT GGA CTC CCC TAG CCC TAC TC	Reverse amplification primer exon 2
CCR7_3F	GAT GAT GCG GAC CTC ACG ATG	Forward amplification primer exon 3
CCR7_3R	CAT GAG GAG AGG TTT TCA GTC CC	Reverse amplification primer exon 3

SeqCCR7_2F	ACA AGA AGG AGG TGA GGA CAG TGA	Sequencing primer exon 2
SeqCCR7_3F_1	GTT GGA GCC ACC CAG CTA AAC TG	Sequencing primer exon 3
SeqCCR7_3F_2	GGG TCT TCG GTG TCC ACT TTT GC	Sequencing primer exon 3
SeqCCR7_3F_3	TGC TCC AGG CAC GCA ACT TTG A	Sequencing primer exon 3
SeqCCR7_3F_4	GCA GAT GCA ATG ACT CAG GAC	Sequencing primer exon 3
SeqCCR7_3F_5	CAG CTG GTC AA ACA AAC TCT C	Sequencing primer exon 3

Primer selection for PCR product amplification and sequencing

### Sequencing

DNA sequencing was carried out using an ABI Prism 3100-Avant genetic analyser (Applied Biosystems) and ABI PRISM BigDye Terminator v1.1 Cycle Sequencing Kit according to the manufactures instructions. Sequence PCR reactions were done for 25 cycles with an annealing temperature of 50°C. The average length of primary sequence was approximately 480 bp. The sequence of the 2.272 bp PCR product spanning exon 3 was revealed applying five internal primers that allowed for an overlap of at least 30 bp between the individual reactions. All polymorphisms detected were confirmed by sequencing of the complementary strand (primer not shown). Primers used are listed in Table 3.

### Cloning of the *CCR7 *promoter constructs and luciferase assays

In order to test whether -60 C/T variant has any functional consequences on CCR7 expression we cloned a putative 563 bp promoter fragment upstream from exon 1. Constructs were amplified from genomic DNA using the Expand High Fidelity PCR System (Roche). For amplification of the mutant promoter bearing the C/T exchange in position -60, genomic DNA of the patient carrying this SNP was used as template (see Table 2 and Fig. 1). The primers used for the amplification (5'-CTC GAG ATC TGA AGG GGG GAG AAA AAA GAT ACA TCG TG-3' and 5'-TGC CAA GCT TGA CGC TCT CTG GGC GGT AAA ACC-3') include 10 additional bases containing cleavage sites for the endonucleases BglII and HindIII respectively. The PCR reaction was performed under standard conditions for 40 cycles with an annealing temperature of 58°C. Subsequent to purification of PCR products via gel extraction (QIAquick gel extraction kit, QIAgen), products were digested for 1 h with HindIII/BglII at 50°C and cloned into the multiple cloning site of a firefly luciferase reporter plasmid (pGL2-basic, Promega). All inserts were checked for correct orientation and sequence.

The functionality of the cloned promoters has been tested by measuring luciferase activity in HUT78 cells and primary T cells transiently transfected with the constructs bearing either the wild type or the mutant promoter. To adjust for transfection efficiency, each construct was co-transfected with a renilla luciferase reporter plasmid. Transfection of HUT78 cells has been performed using a conventional eletroporation protocol. For the transfection of primary T cells, lymphocytes were isolated from 20 ml freshly collected venous blood applying ficoll gradient centrifugation. After washing twice in PBS, lymphocytes were stained with a biotinylated mouse-anti-human CD20 (Caltag) monoclonal antibody followed by streptavidin coated magnetic particles (Miltenyi Biotec, Germany). CD20^+ ^cells were depleted from the cell suspension using the autoMACS cell sorter system (Miltenyi). CD20-negative, non-adherent cells, which were > 85% T cells, were co-transfected with the firefly luciferase reporter plasmid including the promoter construct and a renilla luciferase reporter plasmid (pRL-TK) applying the Human T Cell Nucleofector Kit and the Nucleofector device (AMAXA). In addition, other T cell fractions were co-transfected with the pGL2-basic vector (without any promoter construct) and pRL-TK. For each donor electroporation was done in triplicate. Following electroporation, cells were cultured for at least 24 hrs at 37°C, 5% CO_2_. Luciferase activity in lysates of the transfected cells was determined in a luminometer (Lumat LB 9507, Berthold) using the Dual-Luciferase Reporter Assay System (Promega) according to the manufacture's guidelines. Relative luciferase activity (RLA) was calculated as follows:

RLA=firefly luciferase activity of plasmid with insertrenilla luciferase activity÷firefly luciferase activity of plasmid without insertrenilla luciferase activity
 MathType@MTEF@5@5@+=feaafiart1ev1aaatCvAUfKttLearuWrP9MDH5MBPbIqV92AaeXatLxBI9gBaebbnrfifHhDYfgasaacH8akY=wiFfYdH8Gipec8Eeeu0xXdbba9frFj0=OqFfea0dXdd9vqai=hGuQ8kuc9pgc9s8qqaq=dirpe0xb9q8qiLsFr0=vr0=vr0dc8meaabaqaciaacaGaaeqabaqabeGadaaakeaacqWGsbGucqWGmbatcqWGbbqqcqGH9aqpdaWcaaqaaiabbAgaMjabbMgaPjabbkhaYjabbwgaLjabbAgaMjabbYgaSjabbMha5jabbccaGiabbYgaSjabbwha1jabbogaJjabbMgaPjabbAgaMjabbwgaLjabbkhaYjabbggaHjabbohaZjabbwgaLjabbccaGiabbggaHjabbogaJjabbsha0jabbMgaPjabbAha2jabbMgaPjabbsha0jabbMha5jabbccaGiabb+gaVjabbAgaMjabbccaGiabbchaWjabbYgaSjabbggaHjabbohaZjabb2gaTjabbMgaPjabbsgaKjabbccaGiabbEha3jabbMgaPjabbsha0jabbIgaOjabbccaGiabbMgaPjabb6gaUjabbohaZjabbwgaLjabbkhaYjabbsha0bqaaiabbkhaYjabbwgaLjabb6gaUjabbMgaPjabbYgaSjabbYgaSjabbggaHjabbccaGiabbYgaSjabbwha1jabbogaJjabbMgaPjabbAgaMjabbwgaLjabbkhaYjabbggaHjabbohaZjabbwgaLjabbccaGiabbggaHjabbogaJjabbsha0jabbMgaPjabbAha2jabbMgaPjabbsha0jabbMha5baacqGH3daUdaWcaaqaaiabbAgaMjabbMgaPjabbkhaYjabbwgaLjabbAgaMjabbYgaSjabbMha5jabbccaGiabbYgaSjabbwha1jabbogaJjabbMgaPjabbAgaMjabbwgaLjabbkhaYjabbggaHjabbohaZjabbwgaLjabbccaGiabbggaHjabbogaJjabbsha0jabbMgaPjabbAha2jabbMgaPjabbsha0jabbMha5jabbccaGiabb+gaVjabbAgaMjabbccaGiabbchaWjabbYgaSjabbggaHjabbohaZjabb2gaTjabbMgaPjabbsgaKjabbccaGiabbEha3jabbMgaPjabbsha0jabbIgaOjabb+gaVjabbwha1jabbsha0jabbccaGiabbMgaPjabb6gaUjabbohaZjabbwgaLjabbkhaYjabbsha0bqaaiabbkhaYjabbwgaLjabb6gaUjabbMgaPjabbYgaSjabbYgaSjabbggaHjabbccaGiabbYgaSjabbwha1jabbogaJjabbMgaPjabbAgaMjabbwgaLjabbkhaYjabbggaHjabbohaZjabbwgaLjabbccaGiabbggaHjabbogaJjabbsha0jabbMgaPjabbAha2jabbMgaPjabbsha0jabbMha5baaaaa@0101@

### Statistical analysis

Allelic frequencies were ascertained by direct counting and subsequently analyzed according to the χ^2 ^method. Deviations from Hardy-Weinberg equilibrium were calculated with p-values < 0.05 being considered as significant. Statistical analysis of the promoter activity assay was done using unpaired t-test with p-values < 0.05 being considered significant (GraphPad Prism 4.03; GraphPad Software, San Diego, CA).

## Competing interests

The author(s) declare that they have no competing interests.

## Authors' contributions

DK: performed experiment, drafted manuscript.

ACD-M designed study, wrote manuscript

LO helped to design study and participated in sequence alignment

FS helped to design study

TW: provided DNA samples from patients and controls

RF: designed study, wrote manuscript

## References

[B1] von Andrian UH, Mackay CR (2000). T-cell function and migration. Two sides of the same coin. N Engl J Med.

[B2] Forster R, Schubel A, Breitfeld D, Kremmer E, Renner-Muller I, Wolf E, Lipp M (1999). CCR7 coordinates the primary immune response by establishing functional microenvironments in secondary lymphoid organs. Cell.

[B3] Nakano H, Mori S, Yonekawa H, Nariuchi H, Matsuzawa A, Kakiuchi T (1998). A novel mutant gene involved in T-lymphocyte-specific homing into peripheral lymphoid organs on mouse chromosome 4. Blood.

[B4] Ohl L, Mohaupt M, Czeloth N, Hintzen G, Kiafard Z, Zwirner J, Blankenstein T, Henning G, Forster R (2004). CCR7 governs skin dendritic cell migration under inflammatory and steady-state conditions. Immunity.

[B5] Ohl L, Henning G, Krautwald S, Lipp M, Hardtke S, Bernhardt G, Pabst O, Forster R (2003). Cooperating mechanisms of CXCR5 and CCR7 in development and organization of secondary lymphoid organs. J Exp Med.

[B6] Misslitz A, Pabst O, Hintzen G, Ohl L, Kremmer E, Petrie HT, Forster R (2004). Thymic T cell development and progenitor localization depend on CCR7. J Exp Med.

[B7] Kurobe H, Liu C, Ueno T, Saito F, Ohigashi I, Seach N, Arakaki R, Hayashi Y, Kitagawa T, Lipp M, Boyd RL, Takahama Y (2006). CCR7-dependent cortex-to-medulla migration of positively selected thymocytes is essential for establishing central tolerance. Immunity.

[B8] Davalos-Misslitz AC, Rieckenberg J, Willenzon S, Worbs T, Kremmer E, Bernhardt G, Forster R (2007). Generalized multi-organ autoimmunity in CCR7-deficient mice. Eur J Immunol.

[B9] Hintzen G, Ohl L, del Rio ML, Rodriguez-Barbosa JI, Pabst O, Kocks JR, Krege J, Hardtke S, Forster R (2006). Induction of tolerance to innocuous inhaled antigen relies on a CCR7-dependent dendritic cell-mediated antigen transport to the bronchial lymph node. J Immunol.

[B10] Worbs T, Bode U, Yan S, Hoffmann MW, Hintzen G, Bernhardt G, Forster R, Pabst O (2006). Oral tolerance originates in the intestinal immune system and relies on antigen carriage by dendritic cells. J Exp Med.

[B11] Masi AT (1980). Preliminary criteria for the classification of systemic sclerosis (scleroderma). Subcommittee for scleroderma criteria of the American Rheumatism Association Diagnostic and Therapeutic Criteria Committee. Arthritis Rheum.

[B12] Tan EM, Cohen AS, Fries JF, Masi AT, McShane DJ, Rothfield NF, Schaller JG, Talal N, Winchester RJ (1982). The 1982 revised criteria for the classification of systemic lupus erythematosus. Arthritis Rheum.

[B13] Vitali C (2003). Classification criteria for Sjogren's syndrome. Ann Rheum Dis.

